# Notes and News

**Published:** 1901-01-19

**Authors:** 


### NOTES AND NEWS.

THE prevalence of smallpox in Paris is showing a marked diminution.

THE large new Military Hospital at Portsmouth is to be called after the Princess of Wales, "The Alexandra Hospital."

A TURKISH boatman has died of plague in Constantinople. The Sanitary Council have had a consultation on the appearance of the disease.

LORD GLENESK will preside at the sixty-second annual general meeting of the Newsvendors' Benevolent and Provident Institution on Tuesday, February 12th, at Memorial Hall Buildings, Farringdon Street, at 7 P.M.

A VERY serious outbreak of influenza has appeared in the United States, and from St. Petersburg alarming accounts are sent of the special form the epidemic has taken there. According to reports received death follows the attack with terrible rapidity.

THE Board of Health in New York are about to erect a laboratory especially for the study of bubonic plague. If the United States has seen the advisability of such an institution why with our Indian and other tropical possessions are we without the same advantages in London which educates so many medical men who afterwards work in plague-stricken districts?

IT is highly satisfactory to note that the Metropolitan Hospital Saturday Fund has exceeded last year's collection by upwards of £200 on the present occasion. This result shows, as we predicted it would, that the institution has not suffered by adopting the right course, and abolishing the objectionable system of street collections. The Fund does an admirable work, and its administration is so excellent that it deserves the fullest support and encouragement at the hands of the public. It is a valuable educational force as well as a collecting agency.

Two years ago a female Army doctor was appointed to the United States Army. She was charged with the duties of selecting and equipping a corps of nurses for the army. Her work appears to have been admirably executed, and the lady in question, Dr. A. McGee, has now resigned her post.

Two new Chairs have been created at the Faculty of Medicine in Paris, one being in clinical gynæcology and the other in clinical surgery for the diseases of children.

ATTENTION is being drawn in France to the very serious infant mortality. The industrial and mining districts appear to suffer most, and a connection between the numerous deaths and the large consumption of alcohol is drawn.

THE Local Government Board have sent a letter to the various sanitary authorities stating that they have reason to believe that in a very large number of cases, where the local authorities disinfect or procure the disinfection of premises which have been exposed to infection, carbolic acid is the material employed for the purpose, and warning them that whenever carbolic acid or any other poison within the meaning of the Pharmacy Act, 1868, is employed only bottles similar to those prescribed by the regulations adopted by the Pharmaceutical Society and approved by the Privy Council should be used to contain it.

IT always appears to us that the proper way to deal with the great question of the dwellings of the poor is to take advantage to the full of the powers which are already provided by the law for ensuring that such dwellings shall be maintained in a good sanitary condition. How far our sanitary authorities are from fully performing their duty in this respect is shown by the reports issued from time to time by the Mansion House Council on the Dwellings of the Poor. At the meeting held last week the report which was read showed that the number of houses visited during the month was 580, of which 207 were sent in to the authorities as requiring to be dealt with. It appears as if it were only under the pressure of a private body like this that the public authorities can be induced to keep their districts in full sanitary repair.

AT the annual meeting of the Birmingham Hospital Sunday Fund, the surprising statement was made that the collection of last year only exceeded that made in 1859 by £57. Considering the immense growth of population and wealth in Birmingham, this certainly affords food for reflection to all thoughtful people. Since 1859 the interest in medical charities has been greatly stimulated throughout the British Empire, consistently with the improvements which have taken place both in their medical and domestic condition. It seems strange therefore that, whilst large benefactions have greatly multiplied, a collecting agency, conducted in a satisfactory manner, which permits the charitably-disposed to co-operate in the healing of the sick poor with the minimum of trouble to themselves, should have met with such poor encouragement. It may be argued that the Birmingham Hospital Saturday Fund is so active an institution that it gleans the harvest which the Sunday Fund should reap. But this is not so. The activity of the one fund should stimulate interest in the common cause, and help the collection of the other fund which appeals to quite a different section of the community. We are of opinion that a little of the active spirit which dominates the Hospital Saturday Fund is all that is necessary to remove the present wide difference in the result of the two appealing organisations of the great city of Birmingham.

